# Laparoscopic vs robotic-assisted surgery for treating urachal anomalies in pediatric patients

**DOI:** 10.1007/s00383-026-06434-2

**Published:** 2026-04-27

**Authors:** Ciro Esposito, Fulvia Del Conte, Daniele Alberti, Claudia Di Mento, Marco Castagnetti, Francesco Tedesco, Roberta Guglielmini, Giovanni Boroni, Michele Bosisio, Francesca Carraturo, Tristan Till, Vincenzo Coppola, Giorgia Esposito, Maria Escolino

**Affiliations:** 1 Division of Pediatric Surgery, Federico II University Hospital, Via Pansini, 5, 80131 Naples, Italy; 2 Department of Pediatric Surgery, “Spedali Civili” Children’s Hospital, Brescia, Italy; 3 Division of Pediatric Urology, Bambino Gesù Children Hospital, Rome, Italy; 4 College of Computing and Data Science, Nanyang Technological University, Singapore, Singapore; 5 Department of Internal Medicine, Federico II University Hospital, Naples, Italy

**Keywords:** Urachal anomalies, Urachal cysts, ICG, Laparoscopy, Robotic surgery, Children

## Abstract

**Purpose:**

To report a multicenter experience with minimally invasive surgery (MIS) for pediatric urachal anomalies and to compare laparoscopic versus robotic-assisted approaches.

**Methods:**

A retrospective review was performed of all patients with urachal remnants treated with MIS between January 2019 and January 2025. Demographic, perioperative, and outcome data were collected and analyzed.

**Results:**

Twenty-three patients (13 males) with a median age of 5.9 years (range 6 months–14 years) underwent surgery for symptomatic urachal anomalies. Fifteen patients (65.2%) were treated laparoscopically and eight (34.8%) robotically. Complete excision was achieved in all cases. Median operative time was 45 min (range 33–73). A bladder catheter was maintained for 24 h postoperatively, and all patients were discharged within 48–72 h. Histopathology confirmed urachal remnants without evidence of malignancy. No intraoperative complications occurred. One minor postoperative complication (Clavien–Dindo I) was reported. Median follow-up was 2 years (range 5 months–6 years).

**Conclusion:**

MIS represents the gold standard for the treatment of urachal remnants. Both laparoscopy and robotic-assisted surgery are safe and effective. The robotic approach may offer ergonomic and technical advantages. Indocyanine green (ICG) fluorescence appears to facilitate lesion identification and guide dissection.

## Introduction

The urachus is a three-layered canal that connects the allantois to the fetal bladder, usually obliterates by the fifth month of gestation. After birth it’s represented by a fibrotic cord becoming the median umbilical ligament [1–5].

Urachal abnormalities appear when imperfect obliteration occurs, they can present as a vesical diverticulum (3%), umbilical urachal sinus (18%), urachal cyst (31%) or a patent urachus (48%) [6–9].

Urachal anomalies are rare and often remain asymptomatic. In fact, the true incidence of urachal anomalies is unclear because most urachal remnants remain unknown. Sometimes they are discovered during ultrasound studies performed for recurrent abdominal pain or discovered incidentally during laparoscopic procedure performed for other indications [10–14].

When a urachal remnant becomes inflamed or infected, patients can present fever, umbilical erythema, abdominal/suprapubic pain, hematuria, urinary retention, urinary tract infection, or an abnormal midline mass. Occasionally, patients may have acute abdomen or dyspareunia. Urachal cysts represent most of the pediatric urachal anomaly’s presentation up to 54% [15–18].

In children, minimally invasive surgery (MIS) for urachal anomalies has been limited just to traditional laparoscopic techniques. In recent years, robotic surgery has been increasingly used to treat urinary pathology in children. However, analyzing the international literature, up to date scanty reports of urachal robotic treatment in pediatric patients exist [19–22].

We report a multicenter series of MIS management (laparoscopic and robotic-assisted) of urachal anomalies to evaluate which technique is preferable to adopt in children with this pathology.

## Patients and methods

We conducted a multicentric retrospective study including all consecutive children (0–18 years) who underwent minimally invasive surgery (MIS), either laparoscopic or robotic-assisted, for the treatment of urachal remnants between January 2019 and January 2025. Patients were stratified into two groups according to the surgical approach: Group 1 (G1), laparoscopic approach, and Group 2 (G2), robotic-assisted approach.

Collected data included patient demographics (age, weight), clinical presentation, and preoperative workup (including ultrasound and/or additional imaging when indicated). Surgical variables comprised indication for surgery, operative time (including trocar placement time, docking time for robotic procedures, and total operative time from skin incision to closure), number and size of trocars and/or robotic arms, surgeon experience, intraoperative complications, and need for conversion to open surgery.

Outcome measures included length of hospital stay, postoperative complications within 30 days, need for reintervention, and cosmetic results at follow-up. Postoperative complications were graded according to the Clavien–Dindo classification [23].

### Operative technique

All procedures were performed under general anesthesia with orotracheal intubation and perioperative antibiotic prophylaxis according to institutional protocols. Patients were placed in supine position with slight Trendelenburg tilt. In laparoscopic procedures, a standard three-port technique was used, consisting of one 10-mm umbilical camera trocar and two 5-mm working trocars. In robotic-assisted procedures, the da Vinci Xi system was employed, with three 8-mm robotic trocars and one 5-mm assistant trocar. A 30° laparoscope was used in both approaches.

Indocyanine green (ICG) fluorescence imaging was systematically utilized to enhance visualization of the urachal tract. After insertion of a Foley catheter, the bladder was filled intraoperatively with 20–50 mL of diluted ICG solution. This allowed clear identification of the urachal remnant and its anatomical relationship with the bladder dome.

The urachal remnant was carefully dissected using monopolar hook cautery, proceeding from the umbilicus toward the bladder dome. Particular attention was paid to avoid injury to surrounding structures. Once the bladder insertion was reached, the remnant was excised en bloc, including a small cuff of the bladder dome to ensure complete removal.

**Fig. 1:**
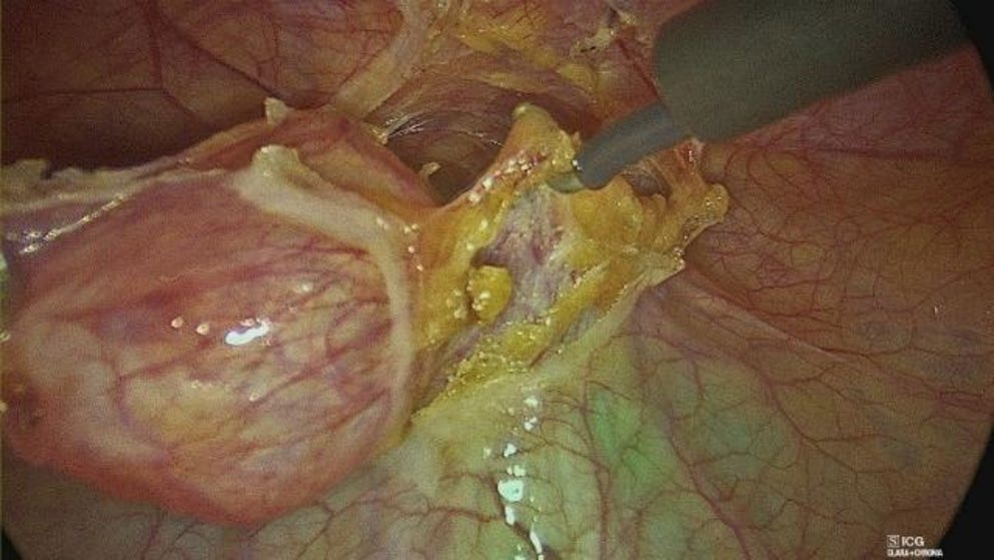
ICG fluorescence guidance in dissection

**Fig. 2:**
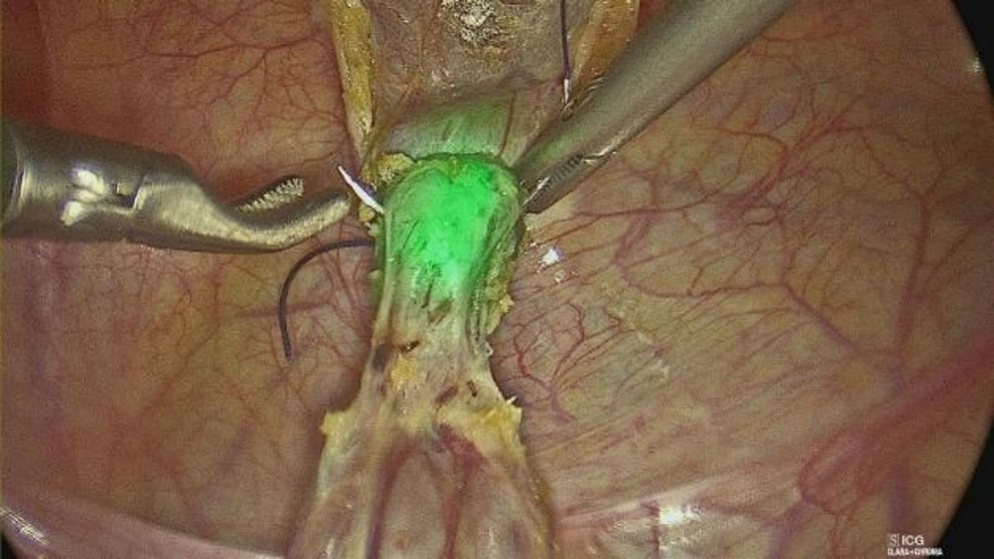
Placement of the trasfixing stich

**Fig. 3:**
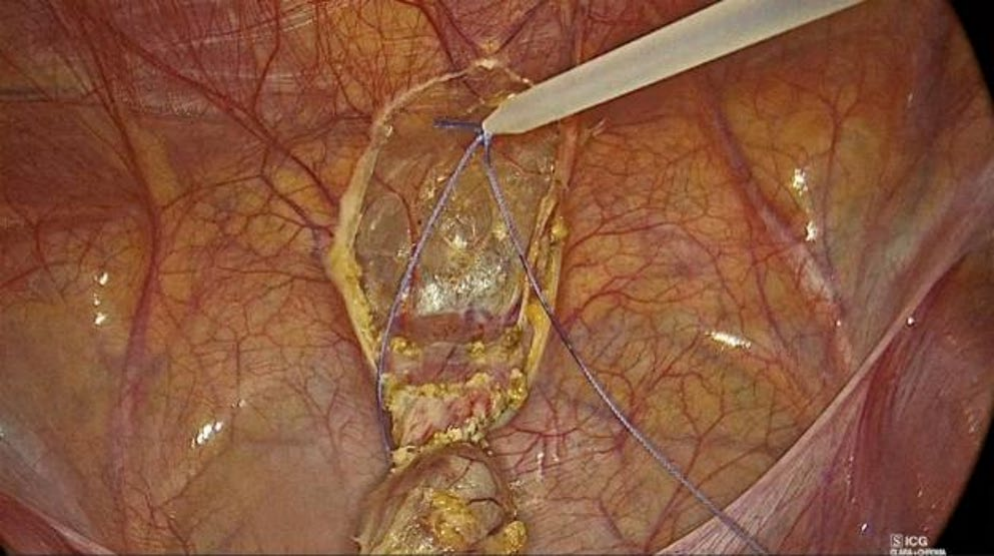
Placement of the endoloop

The bladder defect was closed with a transfixing 2/0 Vicryl suture, reinforced with an endoloop when required. Hemostasis was carefully checked, and no drains were routinely placed (Figs. 1, 2 and 3).

### Statistical analysis

Descriptive statistics were used to summarize patient demographics, surgical variables, and outcomes. Continuous variables are presented as mean with range or median with range, as appropriate, while categorical variables are reported as frequencies and percentages.

Comparisons between the laparoscopic (G1) and robotic-assisted (G2) groups were performed using the Student’s *t*-test or Mann–Whitney *U* test for continuous variables, depending on data distribution, and the chi-square or Fisher’s exact test for categorical variables, as appropriate.

A *p*-value < 0.05 was considered statistically significant. Statistical analyses were performed using SPSS software (version 28.0; IBM Corp., Armonk, NY, USA).

## Results

Between January 2019 and January 2025, a total of 23 patients (13 males, 10 females) underwent MIS for urachal anomalies across participating centers. The median patient age was 5.9 years (range 6 months–14 years) and the median weight was 34.7 kg (range 7.4–87 kg).

Preoperative evaluation included abdominal ultrasonography in all patients, which was sufficient to confirm the diagnosis; no additional imaging was required. All patients presented with symptomatic urachal anomalies. Reported symptoms included recurrent abdominal pain (10 patients, 43%), umbilical discharge (5, 22%), urinary tract infections (6, 26%), and dysuria (9, 39%).

Fifteen patients (65.2%) underwent laparoscopy (G1), with a median age of 4 years (range 6 months-8 years) and median weight of 19.5 kg (range 7.4–37 kg). Eight patients (34.8%) underwent robotic-assisted surgery (G2), with a median age of 10 years (range 7–14 years) and a median weight of 58 kg (range 43–87 kg) (Table 1).

**Table 1:** Patient characteristics

Variable	Value
Total number of patients, n=	23
Sex (M/F), n/n	13/10
Mean age, years (range)	5.9 (6 months – 14 years)
Weight, kg (range)	34.7 (7.4–87)
Laparoscopic approach, n=	15
Robotic approach, n=	8

All procedures were successfully completed using MIS, with no intraoperative complications or conversions to open surgery. Histopathological examination confirmed benign urachal remnants in all cases.

Median operative time was comparable between groups (overall mean 45 min; range 38–70 min in G1 and 33–72 min in G2) [*p* = 0.33]. Docking time was significantly longer in the robotic group: median trocar placement time in laparoscopy was 7 min (range 5–11), while median docking time in robotics was 20 min (range 17–35) [*p* = 0.001].

All robotic procedures were performed using the da Vinci Xi system with a dual console, allowing partial involvement of trainees under senior supervision. Laparoscopic procedures were performed exclusively by senior surgeons.

Postoperatively, all patients had a Foley bladder catheter for 24 h. Median hospital stay was 58 h, being significantly shorter in G2 (52 h) compared to G1 (63 h) [*p* = 0.001].

At a mean follow-up of 2 years (range 5 months–6 years), only one patient (4%) in G1 developed a minor surgical-site infection (Clavien–Dindo I). Cosmetic outcomes were reported as satisfactory in all patients at 2-month follow-up.

Operative and postoperative data are summarized in Table 2.

**Table 2:** Operative and postoperative data

Variable	G1 Laparoscopic (*n* = 15)	G2 Robotic-assisted (*n* = 8)	*p*-value
Median operative time without docking, min (range)	45 (38–70)	45 (33–72)	0.33
Median docking time, min (range)	7 (5–11)	20 (17–35)	0.001
Conversion to open surgery, n= (%)	0	0	-
Intraoperative complications, n= (%)	0	0	-
Median catheter duration, hours (range)	24 (10–36)	24 (12–34)	0.77
Median length of hospital stay, hours (range)	63 (48–72)	52 (36–60)	0.001
Postoperative complications, n= (%)	1 (4) (Clavien–Dindo I)	0	0.89
Median follow-up, months (range)	28 (12–72)	20 (5–60)	0.66
Recurrence	0	0	-
Cosmetic outcome			
Satisfactory, n= (%)	15 (100)	8 (100)	0.58
Unsatisfactory, n= (%)	0	0	-

## Discussion

Open surgical excision has traditionally represented the standard treatment for symptomatic urachal remnants [24–26]. The classical approach involves complete resection of the urachal tract along with a cuff of the bladder dome, aiming to prevent recurrence and reduce the potential risk of malignant transformation later in life. Although urachal carcinoma is rare, particularly in pediatric populations, its association with persistent urachal remnants in adulthood supports the rationale for complete surgical excision [30–32]. Importantly, most urachal anomalies are diagnosed and treated during childhood, when symptoms such as abdominal pain, umbilical discharge, or urinary tract infections become clinically evident [1, 3, 27, 28].

Since the first report of laparoscopic excision of urachal cysts by Trondsen in the early 1990s [29], minimally invasive surgery (MIS) has progressively replaced open surgery in many centers. The advantages of MIS—reduced postoperative pain, shorter hospital stay, faster recovery, and improved cosmetic outcomes—have been well documented in pediatric surgery. Over the last decade, the introduction and rapid expansion of robotic platforms have further expanded the armamentarium of minimally invasive techniques, particularly in pediatric urology. Nevertheless, despite increasing adoption of robotic surgery, reports specifically addressing robotic-assisted excision of urachal anomalies in children remain limited, and comparative data with laparoscopy are still scarce [19].

In this context, our multicenter experience contributes to the growing body of evidence supporting MIS as the preferred approach for symptomatic urachal remnants. In our series, all procedures were successfully completed using a minimally invasive approach, with no conversions to open surgery and no intraoperative complications. These findings are consistent with previously published studies and further confirm the safety and feasibility of MIS for this condition.

From a technical standpoint, we found the use of a 30° optic particularly advantageous in both laparoscopic and robotic procedures. This allowed optimal visualization of the anterior abdominal wall and facilitated dissection in the preperitoneal space, where the urachal tract is located. Precise identification and dissection of the urachal remnant are critical to ensure complete excision and avoid injury to surrounding structures. In all cases, we performed resection of a small cuff of the bladder dome followed by watertight closure with absorbable sutures, in accordance with current surgical principles [30–32].

A central aspect of our study is the comparison between laparoscopic and robotic-assisted approaches. Although both techniques demonstrated comparable operative times and excellent clinical outcomes, some relevant differences emerged. In our practice, patient selection was influenced by body size and weight: smaller children (typically < 15–20 kg) were preferentially treated laparoscopically, while older or heavier patients were more often managed using a robotic approach. This reflects current practice in many pediatric centers, where the size of robotic instruments and port spacing may limit their use in very small patients.

Robotic surgery offers several well-recognized advantages over conventional laparoscopy. The three-dimensional high-definition visualization, tremor filtration, and articulated instruments with seven degrees of freedom significantly enhance surgical precision. These features are particularly beneficial during delicate dissection and intracorporeal suturing, which represent key steps in the excision of urachal remnants. In our experience, the robotic platform facilitated accurate isolation of the urachal tract, especially in cases extending cranially toward the umbilicus, and allowed a more controlled and ergonomic suturing of the bladder dome.

Furthermore, the use of a dual-console robotic system enabled the involvement of trainees in selected steps of the procedure under direct supervision. This aspect is particularly relevant in academic centers, as it allows safe surgical training without compromising patient outcomes. Interestingly, despite trainee participation, no increase in operative time or complication rate was observed in the robotic group, suggesting that robotic surgery may represent a valuable tool for structured surgical education.

On the other hand, laparoscopy remains a highly effective and widely available technique. Its main advantages include the use of smaller instruments, shorter setup time, and significantly lower costs compared to robotic surgery. In our series, trocar placement time in laparoscopy was markedly shorter than docking time in robotic procedures, which remains a well-known limitation of robotic systems. Additionally, laparoscopy may be more suitable in smaller patients, where port size and spacing are critical considerations.

Cost represents a major limiting factor for the widespread adoption of robotic surgery. The high initial investment for robotic platforms, together with the cost of disposable instruments and their limited lifespan, significantly increases the overall procedural cost compared to laparoscopy. However, ongoing technological advancements and the introduction of new robotic systems—including platforms with smaller and reusable instruments—may help reduce these costs in the future, potentially making robotic surgery more accessible.

Another important aspect addressed in our study is the use of indocyanine green (ICG) fluorescence imaging. In all patients, intraoperative instillation of ICG into the bladder allowed clear visualization of the urachal remnant and its anatomical relationship with the bladder dome. This facilitated precise dissection and may have contributed to the absence of intraoperative complications observed in our series. As highlighted in previous reports, ICG fluorescence is a versatile and safe tool with a wide range of applications in pediatric surgery [33, 34]. Its integration into robotic platforms (e.g., Firefly^®^ technology) further enhances its utility, allowing real-time visualization without additional instrumentation.

The management of incidentally discovered urachal remnants remains a topic of debate. While all patients in our series were symptomatic and therefore clearly indicated for surgical treatment, asymptomatic lesions detected during imaging or unrelated surgical procedures are often managed conservatively. Current evidence suggests that observation with clinical and ultrasonographic follow-up may be appropriate in these cases, given the low risk of complications and malignancy in childhood [4, 11, 27].

This study has several limitations. First, its retrospective design and the relatively small sample size limit the strength of the conclusions and the generalizability of the results. Second, the lack of randomization between laparoscopic and robotic approaches introduces a potential selection bias, as patient allocation was influenced by age, weight, and surgeon preference. Third, the procedures were performed in specialized centers with significant experience in MIS and robotics, which may not reflect outcomes in lower-volume settings. Additionally, although follow-up was adequate, longer-term data would be valuable to confirm the durability of outcomes and the absence of late complications. Finally, a formal cost-analysis comparison between the two approaches was not performed and would be relevant for future studies.

In conclusion, our results support the role of MIS as the preferred approach for the treatment of symptomatic urachal anomalies. Both laparoscopic and robotic-assisted approaches are safe and effective, and associated with excellent functional and cosmetic outcomes, low complication rates, and short hospital stay. While laparoscopy remains a cost-effective and reliable technique, robotic surgery offers significant technical and ergonomic advantages that may simplify complex steps such as dissection and suturing. The choice between the two approaches should therefore be individualized, taking into account patient characteristics, surgeon expertise, and resource availability. Additionally, ICG-guided surgery appears to be a valuable adjunct in improving intraoperative visualization.

## Data Availability

The datasets generated and/or analyzed during the current study are available from the corresponding author on reasonable request.
